# 

**Published:** 1870-11

**Authors:** 


### CONTENTS.

ORIGINAL COMMUNICATIONS.

Report of the Committee on Dental Education ..... 461
Report on Operative Dentistry ..... 471
A Case in Practice ..... 486
Cones of Pumice Stone ..... 488

PROCEEDINGS OF SOCIETIES.

Wisconsin State Dental Society ..... 489
American Academy of Dental Science ..... 493

SELECTIONS.

How to Rest the Mind ..... 495

EDITORIAL.

Consequences of Decay of the Teeth ..... 501

